# Supplementary material for: Bovine viral diarrhoea virus loses quasispecies diversity rapidly in culture
Source: Microb Genom. 2020 Mar 11;6(4):e000343. doi: 10.1099/mgen.0.000343 (PMC7276709; doi:10.1099/mgen.0.000343)
Supplement: Supplementary material 1 [file mgen-6-343-s001.pdf]

Supplementary Table 1

Summary data from reference-led NGS assembly of the six datasets (NADL-dT; NADL-B12; MRI103-dT; MRI103-B12; MRI-P3-dT and MRI-P3-B12) using Seqman Ngen (DNASTAR Inc, Madison, WI, USA). All datasets assembled to their respective reference sequence with high quality and good consistency in the placing of read pairs. -dT and -B12 indicate the primer used for cDNA synthesis.

sample	Total reads	Assembled reads	Average Coverage:	Average Quality (Assembled Sequences)	Assembled Pairs:	Pairs Consistent Within a Contig:	Minimum Match Percentage:
NADL-dT	3587795	578995	10708	36	254788	253507	93
NADL-B12	4740735	50141	793	36	10335	10324	93
MRI103-dT	3577799	11492	220	36	5281	5263	93
MRI103-B12	5064217	2459	40	36	310	309	93
MRI-P3-dT	3491349	115739	2024	36	39242	39190	93
MRI-P3-B12	5328689	15096	236	35	2739	2694	93

Supplementary Table 2: Variant base sites within the BVDV MRI103 and NADL genomes, as sequenced directly from serum or culture fluid, or following PCR amplification. Column headings are as follows:

Ref Position	Base position on reference sequence (serum NGS consensus)
Ref Base	Base encoded by reference sequence at this position
Serum Base	Variant base found in serum NGS dataset
Serum SNP %	Frequency of non-consensus base in serum NGS dataset
Serum Depth	Read depth at this position in serum NGS dataset
Serum entropy	Shannon Entropy at this nucleotide position calculated as H= -Σ(freqA*ln(freqA) +freqC*ln(freqC)+freqG*ln(freqG)+freqT*ln(freqT)), where freqN= fractional frequency of the specified base.
Culture Base	Variant base found in culture NGS dataset
Culture SNP %	Frequency of non-consensus base in culture NGS dataset
Culture Depth	Read depth at this position in culture NGS dataset
Culture entropy	Shannon Entropy at this nucleotide position.
Features	BVDV coding regions
S/NS	Nature of substitutions: S, synonymous; NS, non-synonymous; STOP, creating stop codon; NA, not applicable (substitution in untranslated region).
Residue change	Nature of change from consensus residue to altered residue (one-letter amino acid code; * = stop codon)
Serum PCR call	Ambiguous base calls detected in serum based PCR sequence. Bases are indicated as IUPAC ambiguity codes where trace values were similar for each base and as uppercase/lowercase pairs where the uppercase base had higher peak height in all traces of this position.
P3 PCR call	Ambiguous base calls detected in P3 culture based PCR sequence.
P5 PCR call	Ambiguous base calls detected in P5 culture based PCR sequence.
NADL position	Base position on NADL sequence (M 31182)
MRI103 position	Base position on MRI103 reference sequence (when aligned with NADL)
NADL base	Consensus base found in NADL NGS dataset
variant Base	Variant base found in NADL NGS dataset
NADL SNP %	Frequency of non-consensus base in NADL NGS dataset
NADL Depth	Read depth at this position in NADL NGS dataset
NADL entropy	Shannon Entropy at this nucleotide position

Ref Position	Ref Base	Serum Base	Serum SNP %	Serum Depth	Serum entropy		Culture Base	Culture SNP %	Culture Depth	Culture entropy		Features	S/NS	Residue change	Serum PCR call	P3 PCR call	P5 PCR call
41	T						C	2.8%	1945	0.1299		UTR	NA				
58	C						T	1.5%	3239	0.0801		UTR	NA				
67	C	A	1.5%	261	0.0665							UTR	NA				
69	A	C	14.5%	269	0.4104							UTR	NA				
118	G	A	1.0%	416	0.0542							UTR	NA				
120	G	T	7.4%	419	0.2638		T	5.7%	3723	0.2215		UTR	NA				
154	G	A	0.7%	570	0.0418							UTR	NA				
202	C	A	1.3%	752	0.0796							UTR	NA				
203	G	A	0.8%	752	0.0465		A	4.1%	6074	0.1759		UTR	NA				
207	A						G	1.5%	6135	0.0824		UTR	NA				
237	C	T	1.7%	772	0.0814							UTR	NA				
270	G	A	1.0%	714	0.0551							UTR	NA				
277	C						T	1.4%	4155	0.0737		UTR	NA				
278	G	A	0.8%	658	0.0447							UTR	NA				
292	C	T	0.7%	557	0.0426							UTR	NA				
305	T	A	0.9%	567	0.0437							UTR	NA				
384	A	G	1.0%	406	0.0461							Npro	NS	TA			
434	T	A	1.4%	140	0.0749							Npro	NS	DE			
452	G	A	20.5%	156	0.5074		A	8.2%	2473	0.2838		Npro	S				
458	C						T	1.9%	2502	0.0948		Npro	S				
464	T	C	1.3%	152	0.0701							Npro	S				
478	G	A	1.3%	157	0.0682							Npro	NS	RK			
485	A	G	1.2%	171	0.0637							Npro	S				
504	C	T	1.1%	184	0.0600							Npro	S				
509	G						A	1.0%	2843	0.0595		Npro	S				
525	G	A	1.0%	198	0.0565							Npro	NS	GR			

Ref Position	Ref Base	Serum Base	Serum SNP %	Serum Depth	Serum entropy		Culture Base	Culture SNP %	Culture Depth	Culture entropy		Features	S/NS	Residue change	Serum PCR call	P3 PCR call	P5 PCR call
526	G	A	1.0%	203	0.0553							Npro	NS	GE			
531	C	T	1.0%	206	0.0547							Npro	NS	RC			
533	C	G	1.0%	207	0.0544							Npro	S				
536	T	C	1.0%	208	0.0542							Npro	S				
542	C						T	4.2%	2918	0.1822		Npro	S				
558	T	C	1.0%	193	0.0577							Npro	S				
560	G	T	1.5%	200	0.0609							Npro	NS	LF			
584	G	A	0.9%	223	0.0512							Npro	S				
591	G						A	1.1%	2628	0.0590		Npro	NS	GS			
592	G	A	6.4%	220	0.2369		A	4.6%	2641	0.1852		Npro	NS	GD			G/a
626	A	C	0.8%	252	0.0463							Npro	S				
646	A	T	0.7%	286	0.0417							Npro	NS	DV			
653	G						A	6.4%	2541	0.2425		Npro	S				
656	T	G	6.7%	312	0.2466		G	28.2%	2562	0.6249		Npro	S			T/g	
686	C						A	1.4%	3766	0.0751		Npro	S				
692	G	A	0.8%	355	0.0488							Npro	S				
707	T	C	1.4%	361	0.0730							Npro	S				
713	G	A	1.9%	363	0.0952							Npro	S				
767	T						C	2.9%	3865	0.1365		Npro	S				
797	G						A	11.1%	3857	0.3555		Npro	S				
812	T						C	1.3%	3958	0.0702		Npro	S				
815	C	T	1.1%	359	0.0612							Npro	S				
846	C	T	0.9%	329	0.0401							Npro	S				
860	A	G	1.0%	312	0.0419							Npro	S				
866	C	T	0.6%	311	0.0389							Npro	S				
883	C	T	0.7%	301	0.0399							C	NS	TI			

Ref Position	Ref Base	Serum Base	Serum SNP %	Serum Depth	Serum entropy		Culture Base	Culture SNP %	Culture Depth	Culture entropy		Features	S/NS	Residue change	Serum PCR call	P3 PCR call	P5 PCR call
888	G	A	0.7%	299	0.0402							C	NS	DN			
899	A	G	0.8%	249	0.0467							C	S				
902	A	G	1.2%	242	0.0667							C	S				
911	A	G	2.5%	239	0.1173							C	S				
914	A	G	0.8%	244	0.0475							C	S				
929	A	G	0.9%	231	0.0497							C	S				
938	G	A	0.9%	229	0.0501							C	S				
958	C	G	0.8%	245	0.0474							C	NS	PR			
959	C	T	2.4%	248	0.1293							C	S				
979	G	T	1.6%	253	0.0922							C	NS	SI			
983	G	A	1.6%	252	0.0815							C	S				
994	C	T	0.8%	243	0.0477							C	NS	PL			
997	A	T	0.8%	248	0.0469							C	NS	DV			
999	G	T	0.8%	246	0.0472							C	NS	AS			
1007	A	G	0.8%	250	0.0466							C	NS	IM			
1020	G	T	0.8%	250	0.0466							C	NS	VF			
1022	T						C	1.8%	2736	0.0883		C	S				
1028	C	G	0.9%	235	0.0490							C	STOP	Y*			
1029	C	T	1.3%	232	0.0538							C	STOP	Q*			
1033	T	A	1.8%	226	0.0749							C	NS	VE			
1036	A	G	5.9%	220	0.2245							C	NS	KR			
1037	G	A	0.9%	220	0.0518							C	S				
1050	G	A	0.9%	217	0.0524		A	5.7%	2500	0.2181		C	NS	VI			
1054	A	G	1.9%	209	0.0947							C	NS	KR			
1061	A						G	1.1%	2434	0.0610		C	S				
1063	G	A	0.9%	233	0.0494		A	3.8%	2549	0.1681		C	NS	SN			

Ref Position	Ref Base	Serum Base	Serum SNP %	Serum Depth	Serum entropy		Culture Base	Culture SNP %	Culture Depth	Culture entropy		Features	S/NS	Residue change	Serum PCR call	P3 PCR call	P5 PCR call
1070	G	T	1.7%	235	0.0862							C	NS	QH			
1076	T						C	1.3%	2552	0.0730		C	S				
1079	A	G	0.8%	241	0.0480							C	S				
1096	A	G	0.9%	221	0.0516							C	NS	KR			
1100	G	A	2.3%	219	0.1089							C	S				
1102	C	A	1.4%	218	0.0726							C	NS	PQ			
1110	C	T	0.9%	225	0.0508							C	NS	RC			
1112	C						T	1.5%	2633	0.0815		C	S				
1118	A	G	0.9%	227	0.0505							C	S				
1121	T	C	1.8%	225	0.0893							C	S				
1127	A	G	0.9%	212	0.0534							C	S				
1130	A	T	0.9%	223	0.0512							C	S				
1137	G	A	1.4%	222	0.0558							C	NS	AT			
1139	A	T	1.4%	217	0.0569							C	S				
1140	T	A	0.9%	216	0.0526							C	NS	WR			
1155	G	A	24.4%	217	0.5559							C	NS	VM	G/a		
1156	T	C	1.4%	221	0.0718							C	NS	VA			
1159	T	C	1.4%	222	0.0558							C	NS	IT			
1172	T	C	2.2%	230	0.1047							C	S				
1174	C	T	0.9%	230	0.0499							C	NS	TI			
1190	A	T	0.9%	220	0.0518							Erns	S				
1217	G	A	1.0%	202	0.0555							Erns	S				
1220	A						G	1.4%	1718	0.0735		Erns	S				
1234	A	G	1.9%	214	0.0929							Erns	NS	QR			
1237	C	T	2.8%	217	0.1265		T	3.0%	1635	0.1397		Erns	NS	AV			C/t
1243	T	A	0.9%	220	0.0518							Erns	NS	FY			

Ref Position	Ref Base	Serum Base	Serum SNP %	Serum Depth	Serum entropy		Culture Base	Culture SNP %	Culture Depth	Culture entropy		Features	S/NS	Residue change	Serum PCR call	P3 PCR call	P5 PCR call
1244	C	T	0.9%	217	0.0524		T	7.5%	1590	0.2660		Erns	S			C/t	
1246	A	G	0.9%	216	0.0526							Erns	NS	QR			
1257	A	T	17.4%	218	0.6320		T	5.8%	1388	0.2846		Erns	NS	NY			
1258	A	G	1.8%	219	0.1039							Erns	NS	NS			
1259	C	G	11.7%	222	0.4030		G	13.2%	1373	0.4720		Erns	NS	NK	C/g		
1282	C	T	0.9%	223	0.0512							Erns	NS	PL			
1285	A	G	1.3%	234	0.0686							Erns	NS	EG			
1286	A	C	14.1%	234	0.4068		C	40.9%	1108	0.6927		Erns	NS	ED	A/c	A/c	M
1289	A	G	0.9%	235	0.0490							Erns	S				
1293	T	C	1.7%	239	0.0967							Erns	NS	CR			
1295	T	C	9.4%	233	0.3126		C	2.5%	1060	0.1152		Erns	S				
1297	C	T	0.9%	232	0.0496							Erns	NS	TI			
1304	C						T	2.4%	902	0.1147		Erns	S				
1307	T	C	1.9%	214	0.0929							Erns	S				
1312	A	T	1.3%	228	0.0546							Erns	NS	HL			
1332	C						A	2.2%	558	0.0933		Erns	NS	LI			
1338	A	G	9.0%	233	0.3028		G	2.6%	491	0.1223		Erns	NS	TA			
1360	C	T	1.3%	239	0.0674							Erns	NS	AV			
1363	G						C	1.0%	291	0.0574		Erns	NS	ST			
1365	G						A	1.2%	248	0.0509		Erns	NS	EK			
1366	A	G	0.9%	230	0.0499							Erns	NS	EG			
1369	A	G	1.3%	228	0.0701							Erns	NS	KR			
1379	C						T	1.4%	504	0.0665		Erns	S				
1385	T	C	1.7%	242	0.0842							Erns	S				
1386	T	C	0.8%	243	0.0477							Erns	NS	CR			
1388	C	T	0.8%	243	0.0477							Erns	S				

Ref Position	Ref Base	Serum Base	Serum SNP %	Serum Depth	Serum entropy		Culture Base	Culture SNP %	Culture Depth	Culture entropy		Features	S/NS	Residue change	Serum PCR call	P3 PCR call	P5 PCR call
1392	C	T	1.7%	241	0.0845							Erns	S				
1416	C	T	2.3%	215	0.1105							Erns	NS	HY			
1417	A						G	3.0%	807	0.1338		Erns	NS	HR			
1418	T	C	0.9%	216	0.0526							Erns	S				
1436	C	T	1.4%	221	0.0561							Erns	S				
1441	T	A	0.9%	215	0.0528							Erns	NS	IN			
1443	G	A	0.9%	213	0.0532							Erns	NS	EK			
1472	C	T	1.0%	202	0.0555							Erns	S				
1477	C	T	1.0%	203	0.0553							Erns	NS	AV			
1478	T						C	1.6%	1190	0.0819		Erns	S				
1483	T	C	1.8%	167	0.0900							Erns	NS	LP			
1484	T						C	2.1%	1216	0.1010		Erns	S				
1485	A	T	44.9%	167	0.6880		T	82.8%	1218	0.4597		Erns	NS	TS	W	T/a	T/a
1488	G	T	1.1%	176	0.0622							Erns	STOP	E*			
1504	G	A	37.8%	193	0.7107		A	79.4%	1415	0.5091		Erns	NS	RK	R	A/g	A/g
1514	A	G	1.1%	189	0.0587							Erns	S				
1526	G						A	2.7%	1592	0.1242		Erns	S				
1536	G	A	8.2%	195	0.2838		A	1.7%	1686	0.0894		Erns	NS	DN			
1537	A	G	3.6%	196	0.1613							Erns	NS	DG			
1538	T	C	13.2%	197	0.3901		C	8.7%	1693	0.3090		Erns	S		T/c		
1541	T	C	27.6%	196	0.5887							Erns	S				
1543	A	G	3.0%	199	0.1353							Erns	NS	DG			
1545	C	T	3.0%	199	0.1545							Erns	S				
1558	C	T	1.0%	203	0.0553							Erns	NS	TI			
1561	A						T	1.4%	1892	0.0785		Erns	NS	QL			
1564	C	A	1.0%	206	0.0547							Erns	NS	AD			

Ref Position	Ref Base	Serum Base	Serum SNP %	Serum Depth	Serum entropy		Culture Base	Culture SNP %	Culture Depth	Culture entropy		Features	S/NS	Residue change	Serum PCR call	P3 PCR call	P5 PCR call
1574	T	C	1.7%	241	0.0845							Erns	S				
1582	C	T	5.7%	246	0.2184		T	3.8%	2301	0.1599		Erns	NS	PL	C/t		
1596	A	G	1.8%	221	0.1031							Erns	NS	KE			
1602	G	A	1.9%	209	0.0799							Erns	NS	GR			
1603	G	T	1.9%	207	0.0640							Erns	NS	GV			
1604	A	G	1.0%	210	0.0538		G	5.5%	2580	0.2120		Erns	S				
1606	A	G	1.0%	210	0.0538							Erns	NS	KR			
1608	A	T	0.9%	222	0.0514							Erns	NS	NY			
1627	T	C	0.9%	225	0.0508							Erns	NS	IT			
1633	C	T	3.4%	234	0.1490		T	9.5%	3423	0.3145		Erns	NS	SL			
1636	A	T	1.7%	233	0.0987							Erns	NS	QL			
1638	G	A	1.8%	228	0.0883							Erns	NS	GS			
1640	T	C	2.6%	228	0.1097							Erns	S				
1645	G	A	0.9%	230	0.0499							Erns	NS	CY			
1646	C						T	1.1%	3328	0.0614		Erns	S				
1659	G	A	13.7%	234	0.3990		A	9.8%	3313	0.3205		Erns	NS	AT			
1660	C	T	3.0%	234	0.1344		T	2.1%	3313	0.1051		Erns	NS	AV			
1661	T	A	0.9%	235	0.0490							Erns	S				
1663	C						T	1.3%	3298	0.0734		Erns	NS	AV			
1664	G	A	7.6%	236	0.2628		A	8.5%	3295	0.2915		Erns	S				
1691	C	T	1.6%	252	0.0925							Erns	S				
1693	G	T	0.8%	252	0.0463							Erns	NS	CF			
1699	G	A	1.2%	256	0.0638							Erns	NS	SN			
1703	G	A	0.8%	256	0.0457							Erns	S				
1704	T	C	0.8%	256	0.0457							Erns	NS	FL			
1725	C	T	2.0%	247	0.0990							Erns	NS	LF			

Ref Position	Ref Base	Serum Base	Serum SNP %	Serum Depth	Serum entropy		Culture Base	Culture SNP %	Culture Depth	Culture entropy		Features	S/NS	Residue change	Serum PCR call	P3 PCR call	P5 PCR call
1727	T	C	1.2%	254	0.0642							Erns	S				
1730	T	C	0.8%	248	0.0469							Erns	S				
1733	T	C	0.8%	242	0.0479							Erns	S				
1735	G	A	22.4%	241	0.5320		A	7.1%	2813	0.2568		Erns	NS	GE	G/a	G/a	
1737	A	G	13.2%	234	0.4420		G	29.2%	2805	0.6619		Erns	NS	MV		A/g	A/g
1748	C						T	1.8%	2703	0.0938		Erns	S				
1749	T	C	10.0%	221	0.3544		C	3.2%	2697	0.1461		Erns	S				
1752	G						T	1.1%	2673	0.0626		Erns	STOP	E*			
1753	A						C	1.0%	2623	0.0573		Erns	NS	EA			
1758	G	A	0.9%	211	0.0536							Erns	NS	AT			
1767	G	A	1.0%	210	0.0538							Erns	NS	GR			
1793	A						G	1.5%	2240	0.0801		Erns	S				
1809	A	T	2.7%	148	0.1243							Erns	NS	IL			
1811	A	C	1.4%	144	0.0732							Erns	S				
1817	A	G	14.4%	139	0.4120							Erns	S		A/g		
1822	A	G	1.5%	136	0.0766							Erns	NS	KR			
1826	G	A	1.7%	121	0.0842							Erns	S				
1859	T						C	1.4%	217	0.0729		Erns	S				T/c
1868	C						T	5.0%	241	0.1979		E1	S				
1889	A						T	9.6%	335	0.3653		E1	S			A/t	A/t
1900	A	T	1.9%	103	0.0958							E1	NS	YF			
1909	T						A	9.7%	371	0.3149		E1	NS	FY		T/a	
1915	A						G	8.9%	383	0.2997		E1	NS	KR	A/g		A/g
1919	T						G	1.2%	416	0.0566		E1	NS	NK			
1969	A						T	1.7%	586	0.0811		E1	S				
1973	C						T	2.6%	615	0.1206		E1	S				

Ref Position	Ref Base	Serum Base	Serum SNP %	Serum Depth	Serum entropy		Culture Base	Culture SNP %	Culture Depth	Culture entropy		Features	S/NS	Residue change	Serum PCR call	P3 PCR call	P5 PCR call
2044													NS	SF	C/t		
2075	A						G	5.1%	837	0.2025		E1	S				
2086	T						C	7.5%	850	0.2671		E1	NS	VA	T/c		
2104	A	G	2.0%	100	0.0980							E1	NS	HR			
2109	T						A	1.1%	923	0.0652		E1	NS	ST			
2113	T	C	2.0%	100	0.0980							E1	NS	IT			
2117	G	A	2.9%	103	0.1317		A	7.1%	954	0.2569		E1	S				
2123	C	T	1.9%	104	0.0950							E1	S				
2132	T	C	14.9%	101	0.4201		C	7.1%	916	0.2561		E1	S		T/c	T/c	
2138												E1	NS	MI			G/a
2144	C						T	5.5%	981	0.2131		E1	S				
2171	G						A	3.1%	967	0.1383		E1	S				
2180	A	T	1.8%	114	0.0883							E1	S				
2181	A						G	1.3%	948	0.0679		E1	NS	TA			
2189	T						C	2.7%	973	0.1232		E1	S				
2221	G	A	37.4%	115	0.6610		A	4.5%	1016	0.1844		E1	NS	RK			G/a
2225	T						C	2.4%	1023	0.1148		E1	S				
2228	C						T	1.8%	1028	0.0921		E1	S				
2240	G	A	1.7%	116	0.0871							E1	S				
2241	A	G	43.5%	115	0.6846		G	5.9%	960	0.2252		E1	NS	ND	A/g		
2252	T	A	1.7%	116	0.0871							E1	S				
2258	G	A	1.7%	119	0.0853		A	2.0%	957	0.0975		E1	S				
2271	C						A	3.5%	864	0.1480		E1	NS	LM			
2279	C						T	1.1%	945	0.0599		E1	S				
2283	G	T	29.1%	117	0.6027							E1	STOP	E*	G/t		
2284	A	C	28.8%	118	0.6005							E1	NS	EA			

Ref Position	Ref Base	Serum Base	Serum SNP %	Serum Depth	Serum entropy		Culture Base	Culture SNP %	Culture Depth	Culture entropy		Features	S/NS	Residue change	Serum PCR call	P3 PCR call	P5 PCR call
2289	G	A	1.7%	117	0.0865		A	1.2%	895	0.0624		E1	NS	GS			
2296	T	C	1.7%	118	0.0859							E1	NS	VA			
2312	G	A	8.3%	132	0.2868		A	7.5%	939	0.2652		E1	S				
2339	T	C	3.5%	142	0.1524		C	1.1%	1033	0.0590		E1	S				
2343	A	G	27.7%	141	0.5897							E1	NS	ND	A/g		
2352	A	G	23.7%	139	0.5481							E2	NS	TA			
2353	C	T	1.4%	138	0.0758							E2	NS	TM			
2366	T	C	1.4%	146	0.0724							E2	S				
2369	A	G	1.4%	140	0.0749							E2	S				
2378	C						G	1.2%	1004	0.0613		E2	S				
2381	T						C	5.1%	1005	0.2007		E2	S				
2384	G						A	1.3%	996	0.0692		E2	S				
2396	C	T	4.0%	126	0.1669							E2	S				
2421	C						T	2.9%	1126	0.1323		E2	S				
2429	G	A	12.6%	167	0.3782		A	38.9%	1158	0.6686		E2	S			R	R
2441	G						T	1.1%	1126	0.0590		E2	S				
2467	A	G	22.7%	163	0.5356		G	10.1%	1174	0.3281		E2	NS	EG	A/g	A/g	
2470	A						T	5.0%	1248	0.2047		E2	NS	YF			
2486	C	A	1.1%	181	0.0608							E2	S				
2494	A	T	1.1%	176	0.0622							E2	NS	DV			
2495	T						C	2.6%	1361	0.1222		E2	S				
2504	C						T	2.0%	1402	0.0958		E2	S				
2507	A						G	1.2%	1405	0.0605		E2	S				
2525	A						G	7.7%	1606	0.2709		E2	S				
2532	G	A	45.9%	233	0.7325		A	79.4%	1643	0.5082		E2	NS	VI	R	A/g	A/g
2533	T	C	5.6%	232	0.2159		C	3.7%	1649	0.1583		E2	NS	VA			

Ref Position	Ref Base	Serum Base	Serum SNP %	Serum Depth	Serum entropy		Culture Base	Culture SNP %	Culture Depth	Culture entropy		Features	S/NS	Residue change	Serum PCR call	P3 PCR call	P5 PCR call
2538	A	G	1.7%	229	0.0880							E2	NS	KE			
2551	G	A	0.9%	223	0.0512							E2	NS	SN			
2554	G	A	0.9%	223	0.0512							E2	NS	GE			
2560	T	C	22.9%	231	0.5365		C	8.5%	1759	0.2906		E2	NS	VA	T/c		
2564	G						A	2.2%	1946	0.1061		E2	S				
2581	T	G	1.6%	246	0.0831							E2	NS	IR			
2587	G	A	0.8%	240	0.0482							E2	STOP	W*			
2609	A						G	2.0%	2066	0.0993		E2	S				
2628	A	G	9.5%	241	0.3149							E2	NS	RG			
2666	C	T	0.9%	234	0.0492		T	1.6%	1994	0.0807		E2	S				
2668	T	C	1.3%	233	0.0688							E2	NS	LP			
2712	C	A	27.0%	222	0.5835		A	75.9%	1856	0.5675		E2	NS	QK	C/a	A/c	A/c
2729	A	G	4.5%	224	0.1824		G	4.9%	1877	0.2058		E2	S				
2745	G	A	0.9%	228	0.0503							E2	NS	EK			
2748	T	C	1.3%	224	0.0711							E2	NS	FL			
2755	T	A	1.4%	217	0.0729							E2	NS	LH			
2773	A	G	1.0%	208	0.0542							E2	NS	KR			
2776	C	A	1.0%	197	0.0567							E2	NS	PH			
2783	G	A	1.1%	187	0.0592							E2	S				
2798	C						A	1.4%	1614	0.0747		E2	NS	NK			
2807	G						A	1.8%	1651	0.0934		E2	S				
2816	A	T	1.1%	189	0.0587							E2	S				
2817	C	T	20.1%	189	0.5019							E2	NS	PS	C/t		
2820	G	T	12.8%	187	0.3832							E2	NS	AS	G/t		
2821	C	T	1.1%	186	0.0594							E2	S				
2856	T	C	0.8%	236	0.0489							E2	NS	SP			

Ref Position	Ref Base	Serum Base	Serum SNP %	Serum Depth	Serum entropy		Culture Base	Culture SNP %	Culture Depth	Culture entropy		Features	S/NS	Residue change	Serum PCR call	P3 PCR call	P5 PCR call
2871	T						G	1.4%	1969	0.0724		E2	NS	LV			
2873													S				A/g
2877	A	G	0.9%	223	0.0512							E2	NS	ND			
2881	G	A	1.3%	224	0.0711							E2	NS	RK			
2898	G	A	1.4%	213	0.0740							E2	NS	AT			
2899	C	T	4.7%	215	0.1881							E2	NS	AV			
2900	A	G	1.9%	215	0.0926							E2	S				
2904	A	G	21.3%	225	0.5183		G	9.4%	1963	0.3109		E2	NS	IV	A/g		
2912	G	A	0.9%	234	0.0492							E2	S				
2922	T	A	13.3%	240	0.4677		A	32.5%	2027	0.6303		E2	NS	ST	T/a	T/a	W
2930	A	G	1.3%	234	0.0686							E2	S				
2936	C	T	9.7%	227	0.3123		T	4.3%	1941	0.1852		E2	S				
2954	C	A	8.3%	205	0.2859		A	32.6%	1778	0.6314		E2	S		C/a	C/a	M
2958	C	T	1.9%	210	0.0943							E2	STOP	Q*			
2960	A	G	8.1%	210	0.2811		G	6.9%	1790	0.2494		E2	S				
2962	A	T	0.9%	211	0.0536							E2	NS	KM			
2966	T	C	2.8%	211	0.1167							E2	S				
2981	C	A	1.1%	189	0.0587							E2	S				
2984	C						T	3.1%	1707	0.1384		E2	S				
2985	G	A	1.0%	192	0.0579							E2	NS	DN			
2987	C	A	2.1%	194	0.1004							E2	S				
2998	G	A	1.0%	199	0.0562							E2	NS	GE			
3017	G	A	1.0%	203	0.0553							E2	S				
3048	T	C	1.4%	210	0.0749							E2	NS	SP			
3049	C	T	0.9%	211	0.0536							E2	NS	SF			
3083	T						C	1.8%	1138	0.0920		E2	S				

Ref Position	Ref Base	Serum Base	Serum SNP %	Serum Depth	Serum entropy		Culture Base	Culture SNP %	Culture Depth	Culture entropy		Features	S/NS	Residue change	Serum PCR call	P3 PCR call	P5 PCR call
3084	C	A	1.0%	206	0.0547							E2	NS	QK			
3091	G	A	7.8%	206	0.2730		A	2.6%	926	0.1174		E2	NS	SN			
3092	T	A	8.1%	209	0.2747		A	1.9%	924	0.0928		E2	NS	SR			
3093	G	A	1.0%	199	0.0562							E2	NS	EK			
3101	A						G	1.5%	1019	0.0735		E2	S				
3106	A						T	1.6%	1176	0.0881		E2	NS	HL			
3117	G	A	1.2%	257	0.0494							E2	NS	GS			
3122	G	A	16.9%	260	0.4547		A	32.5%	1534	0.6595		E2	S		G/a	R	R
3132	G	A	0.8%	257	0.0455							E2	NS	EK			
3133	A	T	1.7%	238	0.0853							E2	NS	EV			
3136	A	G	1.3%	238	0.0677							E2	NS	NS			
3137	T	A	0.8%	238	0.0485							E2	NS	NK			
3146	C	T	0.8%	258	0.0454							E2	S				
3151	G	A	0.8%	257	0.0455							E2	NS	RK			
3162	G	A	22.8%	254	0.5373		A	4.4%	3833	0.1844		E2	NS	DN	G/a		G/a
3174	A	G	18.0%	261	0.4715		G	6.6%	3882	0.2488		E2	NS	ND	A/g		
3177	A	G	0.8%	261	0.0450							E2	NS	RG			
3182	A	T	2.3%	261	0.1095		T	9.4%	3918	0.3171		E2	NS	ED		A/t	
3184	G	A	0.8%	261	0.0450							E2	NS	GD			
3186	G	A	0.8%	261	0.0450							E2	NS	VM			
3194	A	G	1.5%	260	0.0795							E2	NS	IM			
3197	A						G	1.6%	3914	0.0834		E2	S				
3200	G	A	0.8%	263	0.0447							E2	S				
3201	C	T	0.8%	262	0.0448							E2	NS	HY			
3203	C	A	18.7%	262	0.4819							E2	NS	HQ	C/a		
3207	C	A	17.2%	262	0.4587		A	6.4%	3901	0.2397		E2	NS	LM	C/a		

Ref Position	Ref Base	Serum Base	Serum SNP %	Serum Depth	Serum entropy		Culture Base	Culture SNP %	Culture Depth	Culture entropy		Features	S/NS	Residue change	Serum PCR call	P3 PCR call	P5 PCR call
3212	A	T	0.8%	265	0.0444							E2	S				
3227	A	C	2.3%	256	0.1260							E2	S				
3232	C	A	1.2%	253	0.0644							E2	NS	TK			
3247	C	T	22.9%	236	0.5378		T	4.4%	3878	0.1828		E2	NS	TI	C/t		C/t
3260	T	A	1.4%	218	0.0567							E2	S				
3262	A	G	0.9%	219	0.0520							E2	NS	KR			
3278	T	A	0.9%	215	0.0528							E2	S				
3284	G	A	1.4%	207	0.0758							E2	S				
3287	A						G	1.4%	3892	0.0731		E2	S				
3288	T	C	16.3%	203	0.4439		C	2.7%	3891	0.1246		E2	NS	YH	T/c		T/c
3298	T	C	3.1%	191	0.1396							E2	NS	NS	IT		T/c
3315	G	A	1.2%	171	0.0637		A	3.3%	3714	0.1483		E2	NS	VI			
3326	G	T	1.2%	166	0.0652							E2	S				
3336	T	G	3.1%	161	0.1384		G	1.0%	3588	0.0564		E2	NS	FV			
3337	T	A	2.5%	161	0.1163							E2	NS	FY			
3341	C	T	1.9%	107	0.0929							E2	S				
3344	C						T	1.0%	2046	0.0609		E2	S				
3354	T						G	1.0%	966	0.0628		E2	NS	LV			
3355	T						C	1.5%	940	0.0815		E2	NS	LS			
3356	G						A	1.0%	884	0.0578		E2	S				
3366	T						A	13.5%	585	0.4322		E2	NS	YN			
3368	T						A	1.9%	520	0.0893		E2	STOP	Y*			
3401	C						T	3.5%	546	0.1510		E2	S				
3411	G						A	1.3%	541	0.0628		E2	NS	GR			
3419	T						C	3.1%	554	0.1371		E2	S				
3420	C						T	1.3%	557	0.0750		E2	STOP	Q*			

Ref Position	Ref Base	Serum Base	Serum SNP %	Serum Depth	Serum entropy		Culture Base	Culture SNP %	Culture Depth	Culture entropy		Features	S/NS	Residue change	Serum PCR call	P3 PCR call	P5 PCR call
3442	C						T	1.9%	531	0.0935		E2	NS	AV			
3468	G						T	2.3%	570	0.1088		E2	NS	AS			
3473												E2	NS	ED	G/t		
3477	A						G	1.4%	552	0.0758		E2	NS	IV			
3479	A						T	1.8%	554	0.0904		E2	S				
3488	A						G	1.1%	564	0.0589		E2	NS	IM			
3491												E2	S		G/a		
3565	C						T	3.6%	667	0.1550		E2	NS	SL			
3622	A						G	8.2%	643	0.2823		p7	NS	DG	A/g		
3648	T						C	9.2%	638	0.3082		p7	S				
3667	T						A	1.3%	706	0.0584		p7	NS	LQ			
3689	G						A	1.6%	749	0.0821		p7	S				
3698												p7	S		G/a		
3714												p7	S		C/t		
3716	A						G	6.3%	827	0.2348		p7	S			A/g	A/g
3753	T						C	1.3%	851	0.0752		p7	S				
3766	A	G	1.9%	107	0.0929							p7	NS	DG			
3768	G	A	5.5%	109	0.2131							p7	NS	VM			
3769	T						C	2.5%	831	0.1146		p7	NS	VA			
3775	A						G	1.2%	892	0.0665		p7	NS	KR			
3787	G	T	1.6%	122	0.0836							NS2	NS	GV			
3790	A	G	22.4%	125	0.5278							NS2	NS	DG	A/g		
3795	G	T	1.6%	125	0.0820							NS2	NS	GW			
3801	T						C	1.8%	1023	0.0947		NS2	S				
3802	T	C	3.1%	127	0.1399							NS2	NS	LS			
3803	G						A	2.3%	1028	0.1081		NS2	S				

Ref Position	Ref Base	Serum Base	Serum SNP %	Serum Depth	Serum entropy		Culture Base	Culture SNP %	Culture Depth	Culture entropy		Features	S/NS	Residue change	Serum PCR call	P3 PCR call	P5 PCR call
3812	A						G	3.6%	1034	0.1520		NS2	NS	IM			
3831	G	A	2.6%	156	0.1192							NS2	NS	VI			
3833	T	A	1.3%	151	0.0704							NS2	S				
3842	C	A	1.3%	160	0.0672							NS2	S				
3843	G	T	1.3%	160	0.0672							NS2	NS	VF			
3857	C	T	1.2%	164	0.0659							NS2	S				
3860	A						G	1.3%	1240	0.0770		NS2	NS	IM			
3882	G	A	1.1%	177	0.0619		A	1.8%	1305	0.0894		NS2	NS	VI			
3891	G	A	8.1%	173	0.2810		A	6.6%	1287	0.2506		NS2	NS	VI			
3902	A	G	1.2%	167	0.0649							NS2	S				
3934	C						T	1.4%	1234	0.0700		NS2	NS	AV			
3936												NS2	NS	PS			C/t
3959	A						G	2.9%	1257	0.1300		NS2	S				
3967	C	G	1.3%	159	0.0675							NS2	NS	TS			
3968	C	A	1.2%	162	0.0665							NS2	S				
4004	C	T	1.4%	143	0.0736							NS2	S				
4048	C	T	1.5%	132	0.0785							NS2	NS	SL			
4066	G	A	7.0%	128	0.2411		A	28.9%	1067	0.6123		NS2	S		G/a	R	G/a
4075	C	T	6.2%	129	0.2325		T	2.6%	1069	0.1212		NS2	S				C/t
4083	G						A	1.4%	1074	0.0735		NS2	NS	GS			
4100	A	G	1.8%	113	0.0889							NS2	S				
4104	G	C	1.6%	126	0.0815							NS2	NS	VL			
4112	C	T	1.5%	133	0.0780							NS2	S				
4134	C	T	1.6%	125	0.0820							NS2	S				
4136	A	G	1.6%	125	0.0820							NS2	S				
4143	C	T	1.7%	117	0.0865							NS2	S				

Ref Position	Ref Base	Serum Base	Serum SNP %	Serum Depth	Serum entropy		Culture Base	Culture SNP %	Culture Depth	Culture entropy		Features	S/NS	Residue change	Serum PCR call	P3 PCR call	P5 PCR call
4147	A	T	1.6%	127	0.0810							NS2	NS	YF			
4151	G	T	1.6%	123	0.0831							NS2	S				
4161	A	G	1.5%	136	0.0766							NS2	NS	TA			
4164	G						A	1.6%	1141	0.0811		NS2	NS	VI			
4174	T	G	3.8%	133	0.1602		G	3.3%	1152	0.1429		NS2	NS	MR			
4195	G	A	1.4%	143	0.0736							NS2	NS	GD			
4202	G	A	1.4%	142	0.0740							NS2	S				
4203	T						C	5.9%	1251	0.2232		NS2	S				
4212	A	G	25.0%	144	0.5623		G	39.0%	1248	0.6689		NS2	NS	MV	A/g	R	G/a
4217	A						G	1.3%	1264	0.0713		NS2	S				
4224	C	T	1.4%	144	0.0732							NS2	S				
4226	G	T	1.4%	144	0.0732		T	3.5%	1273	0.1503		NS2	S				
4234	T	C	28.4%	141	0.5964		C	1.3%	1261	0.0714		NS2	NS	IT	Y		
4241	A	G	1.4%	139	0.0753							NS2	S				
4247	C	A	2.8%	141	0.1290							NS2	S				
4256	A	G	2.8%	144	0.1269		G	2.5%	1194	0.1174		NS2	S				
4263	A	G	21.5%	144	0.5209		G	35.2%	1169	0.6489		NS2	NS	IV	A/g	R	R
4275	C	T	2.9%	140	0.1297							NS2	NS	PS			
4280	C	T	1.4%	138	0.0758							NS2	S				
4283	T	C	1.4%	142	0.0740		C	4.2%	1131	0.1756		NS2	S				T/c
4298	A	G	4.0%	149	0.1534							NS2	S				
4305	C	T	2.7%	149	0.1236		T	8.9%	1113	0.3089		NS2	S			C/t	C/t
4314	A	G	1.3%	149	0.0712		G	4.9%	1107	0.1949		NS2	NS	IV			
4326	A						G	1.6%	1162	0.0835		NS2	NS	IV			
4359	A	C	3.7%	164	0.1802							NS2	NS	KQ			
4367	T	C	1.2%	171	0.0637							NS2	S				

Ref Position	Ref Base	Serum Base	Serum SNP %	Serum Depth	Serum entropy		Culture Base	Culture SNP %	Culture Depth	Culture entropy		Features	S/NS	Residue change	Serum PCR call	P3 PCR call	P5 PCR call
4406	A	T	1.0%	194	0.0574							NS2	S				
4412	T						A	1.9%	1289	0.0903		NS2	S				
4436	G	A	2.2%	185	0.1043							NS2	S				
4450	T	A	1.1%	189	0.0587							NS2	NS	IK			
4466	C	G	1.1%	190	0.0584							NS2	S				
4472	G						A	1.0%	1643	0.0576		NS2	S				
4478	G						A	1.1%	1673	0.0621		NS2	S				
4487	T	C	1.0%	195	0.0572							NS2	S				
4506	T	C	17.5%	189	0.4631		C	30.1%	1822	0.6115		NS2	S			T/c	T/c
4508	A	G	1.1%	189	0.0587							NS2	S				
4541	A	G	2.3%	172	0.1105							NS2	NS	IM			
4556	G						A	1.6%	1702	0.0815		NS2	S				
4562	T	C	2.5%	157	0.1187							NS2	S				
4565	A						C	2.3%	1707	0.1089		NS2	S				
4572	A	G	13.3%	150	0.3927		G	41.2%	1691	0.6776		NS2	NS	IV		A/g	R
4574	C	T	29.5%	149	0.6068							NS2	S				
4591	T	A	1.4%	146	0.0724							NS2	NS	MK			
4604	A	G	7.4%	135	0.2641		G	24.8%	1656	0.5733		NS2	S			R	
4610	T	C	1.6%	127	0.0810							NS2	S				
4622	G	T	3.4%	116	0.1739							NS2	NS	ED			
4641	G						A	1.9%	996	0.1042		NS2	NS	EK			
4647	G						A	1.6%	898	0.0897		NS2	NS	EK			
4700	A						G	5.0%	968	0.1973		NS2	S			A/g	
4701	A	G	1.9%	108	0.0922		G	2.2%	969	0.1015		NS2	NS	IV			
4706	A	G	3.8%	106	0.1607							NS2	NS	IM			
4715	A						G	1.3%	985	0.0702		NS2	S				

Ref Position	Ref Base	Serum Base	Serum SNP %	Serum Depth	Serum entropy		Culture Base	Culture SNP %	Culture Depth	Culture entropy		Features	S/NS	Residue change	Serum PCR call	P3 PCR call	P5 PCR call
4739	G						A	2.7%	1205	0.1226		NS2	S				
4750	G	A	3.2%	124	0.1425							NS2	NS	GE			
4763	C						T	5.3%	1165	0.2062		NS2	S				
4766	C	T	3.0%	135	0.1335							NS2	S				
4772	G	A	1.5%	135	0.0771							NS2	S				
4776	A						G	1.3%	1199	0.0709		NS2	NS	IV			
4778	C	A	1.5%	133	0.0780							NS2	S				
4806	A	T	1.4%	142	0.0740							NS2	NS	NY			
4820	C	T	1.3%	149	0.0712							NS2	S				
4827	A	G	1.3%	152	0.0701							NS2	NS	IV			
4874	A						T	2.9%	1566	0.1303		NS2	S				
4880	T	C	2.5%	162	0.1329							NS2	S				
4889	T	A	1.8%	163	0.0722							NS2	NS	HQ			
4902	A	T	1.2%	166	0.0652							NS2	NS	TS			
4919	A	G	1.9%	159	0.0936							NS2	S				
4936	G						A	1.1%	1321	0.0687		NS2	NS	RK			
4967	C	T	1.4%	143	0.0736							NS2	S				
4978	G	A	1.5%	135	0.0771							NS2	NS	GD			
4995	T						A	1.1%	1128	0.0637		NS2	NS	YN			
5000	T	C	1.5%	132	0.0785							NS2	S				
5015	T	C	1.6%	126	0.0815		C	3.7%	1116	0.1574		NS2	S				
5018	C	T	1.6%	127	0.0810							NS2	S				
5056	T	C	1.6%	124	0.0826							NS2	NS	LP			
5072	A						G	3.9%	767	0.1651		NS2	S				
5076												NS2	NS	LV			C/g
5177	G						A	1.3%	544	0.0688		NS3	S				

Ref Position	Ref Base	Serum Base	Serum SNP %	Serum Depth	Serum entropy		Culture Base	Culture SNP %	Culture Depth	Culture entropy		Features	S/NS	Residue change	Serum PCR call	P3 PCR call	P5 PCR call
5237	T						C	2.8%	499	0.1279		NS3	S				
5267	G						A	1.0%	579	0.0577		NS3	S				
5275	T						A	1.9%	589	0.0928		NS3	NS	LQ			
5304	T						G	1.2%	598	0.0637		NS3	NS	WG			
5319	C						A	1.8%	610	0.0903		NS3	NS	QK			
5327	G						A	2.5%	610	0.1109		NS3	S				
5339	T						C	2.8%	605	0.1337		NS3	S				
5343	C						A	1.4%	584	0.0666		NS3	NS	HN			
5347	T						A	2.5%	590	0.1284		NS3	NS	VE			
5375	C						T	1.6%	964	0.0802		NS3	S				
5432	G						A	1.6%	1144	0.0809		NS3	S				
5459	A						C	1.6%	1122	0.0947		NS3	S				
5477	C	T	1.9%	105	0.0943							NS3	S				
5499	G	T	1.9%	103	0.0958							NS3	STOP	E*			
5504	A	T	1.9%	103	0.0958							NS3	S				
5519	G						A	3.3%	1138	0.1480		NS3	S				
5522	C						A	3.0%	1160	0.1310		NS3	S				
5525	A	G	1.9%	104	0.0950							NS3	S				
5531	A	G	1.9%	106	0.0936							NS3	S				
5539	A	G	2.0%	100	0.0980							NS3	NS	HR			
5561	A	G	1.8%	112	0.0896							NS3	S				
5567	G	A	1.8%	114	0.0883							NS3	S				
5585	C	A	1.8%	111	0.0902							NS3	S				
5588	A	T	1.6%	122	0.0836							NS3	S				
5591	G	C	1.6%	123	0.0831							NS3	S				
5633	G						A	4.8%	1178	0.1912		NS3	S				

Ref Position	Ref Base	Serum Base	Serum SNP %	Serum Depth	Serum entropy		Culture Base	Culture SNP %	Culture Depth	Culture entropy		Features	S/NS	Residue change	Serum PCR call	P3 PCR call	P5 PCR call
5654	C						T	1.8%	1246	0.0942		NS3	S				
5655	G	T	1.6%	126	0.0815							NS3	STOP	G*			
5684	G	A	1.5%	134	0.0776							NS3	S				
5705	C	T	1.5%	130	0.0795							NS3	S				
5720	C	T	3.4%	118	0.1480		T	2.8%	1512	0.1269		NS3	S				
5723	C	T	2.3%	132	0.1085							NS3	S				
5730	A	G	1.5%	135	0.0771							NS3	NS	TA			
5777	A	T	20.7%	188	0.5106		T	4.5%	1861	0.1828		NS3	S				
5813	T	C	1.0%	192	0.0579							NS3	S				
5817	G	T	2.1%	194	0.1004							NS3	NS	AS			
5822	A	G	2.1%	195	0.1000							NS3	S				
5828	A	G	1.0%	194	0.0574							NS3	S				
5831	A						G	2.8%	1845	0.1264		NS3	S				
5834	A						G	3.5%	1834	0.1519		NS3	S				A/g
5836	C						A	1.0%	1831	0.0577		NS3	NS	TN			
5837	C						A	1.1%	1833	0.0727		NS3	S				
5851	A	C	0.9%	211	0.0536							NS3	NS	KT			
5868	A	G	2.0%	205	0.0961							NS3	NS	IV			
5876	A	G	0.9%	213	0.0532							NS3	S				
5891	G	A	0.9%	231	0.0497							NS3	S				
5894	C	T	2.6%	235	0.1188							NS3	S				
5900	A	T	0.9%	233	0.0494							NS3	S				
5903	A						G	2.3%	1846	0.1086		NS3	S				
5915	G	A	0.8%	239	0.0484							NS3	S				
5917	C	T	0.8%	236	0.0489							NS3	NS	AV			
5927	C	T	0.8%	239	0.0484							NS3	S				

Ref Position	Ref Base	Serum Base	Serum SNP %	Serum Depth	Serum entropy		Culture Base	Culture SNP %	Culture Depth	Culture entropy		Features	S/NS	Residue change	Serum PCR call	P3 PCR call	P5 PCR call
5999	C	T	1.0%	209	0.0540							NS3	S				
6000	A	G	1.0%	207	0.0544							NS3	NS	MV			
6003	G	A	1.0%	205	0.0549							NS3	NS	AT			
6008	C						T	1.4%	1975	0.0759		NS3	S				
6016	C	A	1.0%	195	0.0572							NS3	NS	TN			
6029	T	A	1.0%	193	0.0577							NS3	STOP	Y*			
6044	A						G	2.0%	1815	0.1038		NS3	S				
6050	T	C	1.2%	162	0.0665							NS3	S				
6080	A	G	1.3%	153	0.0697							NS3	S				
6083	C	T	9.4%	149	0.3116		T	3.8%	1715	0.1618		NS3	S				
6089	C	T	16.9%	142	0.4543		T	9.4%	1630	0.3106		NS3	S				
6104	A	G	1.5%	130	0.0795							NS3	S				
6212	C						T	26.4%	1213	0.5767		NS3	S		C/t	C/t	C/t
6299	T	A	2.7%	110	0.1251		A	8.3%	1318	0.2842		NS3	S				T/a
6320	G	A	1.8%	112	0.0896							NS3	S				
6328	T	A	1.6%	123	0.0831							NS3	NS	MK			
6359	G	A	23.8%	147	0.5489							NS3	S		G/a		
6377	G	A	1.3%	150	0.0708							NS3	S				
6385	A	G	1.3%	158	0.0679							NS3	NS	KR			
6389	A						G	1.6%	1772	0.0835		NS3	S				
6395	A	G	1.3%	156	0.0686							NS3	S				
6424	A	G	1.2%	170	0.0640							NS3	NS	YC			
6431	G	A	1.1%	176	0.0622							NS3	S				
6437	C	T	1.1%	174	0.0628							NS3	S				
6457	T	C	1.1%	175	0.0625							NS3	NS	VA			
6460	C						T	1.2%	1840	0.0629		NS3	NS	TI			

Ref Position	Ref Base	Serum Base	Serum SNP %	Serum Depth	Serum entropy		Culture Base	Culture SNP %	Culture Depth	Culture entropy		Features	S/NS	Residue change	Serum PCR call	P3 PCR call	P5 PCR call
6461	A						G	1.1%	1847	0.0603		NS3	S				
6464	A						G	2.2%	1926	0.1070		NS3	S				
6474	T	G	1.1%	184	0.0600							NS3	NS	YD			
6480	A	G	27.8%	187	0.5911							NS3	NS		A/g		
6482	T	C	1.1%	187	0.0592							NS3	S				
6485	G	A	1.1%	188	0.0589							NS3	S				
6488	C	A	1.1%	189	0.0587							NS3	S				
6509	A	T	1.1%	186	0.0594							NS3	S				
6519	C	G	1.0%	195	0.0572							NS3	NS	PA			
6525	T	C	1.1%	188	0.0589							NS3	S				
6539	G	A	1.1%	185	0.0597		A	1.5%	1858	0.0782		NS3	S				
6545	A	C	1.1%	185	0.0597							NS3	S				
6547	G	T	1.1%	190	0.0584							NS3	NS	GV			
6579	T	C	2.6%	196	0.1188							NS3	NS	SP			
6585	A	G	1.1%	184	0.0600		G	1.6%	1750	0.0802		NS3	NS	IV			
6590	C	T	3.2%	189	0.1610							NS3	S				
6606	C	T	1.0%	191	0.0582							NS3	NS	LF			
6620	T	C	3.7%	190	0.1578							NS3	S				
6636	C	A	1.1%	178	0.0616							NS3	NS	QK			
6638	G	A	2.7%	185	0.1096							NS3	S				
6644	A						G	1.6%	1773	0.0812		NS3	S				
6659	A	T	2.0%	197	0.1133							NS3	S				
6662	T	C	2.0%	199	0.0831							NS3	S				
6692	C	T	1.6%	188	0.0819							NS3	S				
6710	G	A	1.0%	197	0.0567							NS3	S				
6779	A	G	0.9%	220	0.0518							NS3	S				

Ref Position	Ref Base	Serum Base	Serum SNP %	Serum Depth	Serum entropy		Culture Base	Culture SNP %	Culture Depth	Culture entropy		Features	S/NS	Residue change	Serum PCR call	P3 PCR call	P5 PCR call
6782	A	G	5.3%	227	0.2307		G	1.9%	1790	0.0924		NS3	S				
6808	G	A	0.9%	219	0.0520							NS3	STOP	W*			
6824	G	A	0.9%	212	0.0534		A	6.1%	1792	0.2424		NS3	S				
6833	G	A	1.9%	208	0.0950							NS3	S				
6836	A	G	1.0%	208	0.0542							NS3	S				
6855	C	T	3.4%	204	0.1494							NS3	S				
6860	C						T	2.6%	1683	0.1189		NS3	S				
6877	A	G	1.0%	194	0.0574							NS3	NS	EG			
6878	A	T	2.1%	193	0.1008							NS3	NS	ED			
6881	C	A	4.1%	194	0.1719							NS3	NS	DE			
6882	T	C	2.1%	195	0.1000							NS3	S				
6885	C	T	3.0%	198	0.1358							NS3	NS	PS			
6911	C						T	2.4%	1734	0.1187		NS3	S				
6916	C	T	1.1%	178	0.0616							NS3	NS	TI			
6926	A	G	1.2%	168	0.0646							NS3	S				
6932	G	A	1.8%	163	0.0722							NS3	S				
6950	C						T	2.1%	1799	0.1002		NS3	S				
6971	A	G	1.2%	163	0.0662							NS3	S				
6988	T						A	1.0%	1679	0.0634		NS3	NS	IK			
6998	A	G	6.2%	145	0.2326							NS3	S				
7007	A	G	5.8%	137	0.2225		G	3.8%	1433	0.1605		NS3	S				
7016	C						T	1.7%	1498	0.0923		NS3	S				
7049	A						G	5.2%	1508	0.1996		NS3	S				
7052	A	G	1.4%	146	0.0724							NS3	S				
7058	T	G	1.4%	144	0.0732							NS3	NS	DE			
7071	A	G	1.2%	171	0.0637		G	3.2%	1792	0.1410		NS3	NS	IV			

Ref Position	Ref Base	Serum Base	Serum SNP %	Serum Depth	Serum entropy		Culture Base	Culture SNP %	Culture Depth	Culture entropy		Features	S/NS	Residue change	Serum PCR call	P3 PCR call	P5 PCR call
7088	T	C	2.3%	172	0.1105							NS3	S				
7095	C	A	1.2%	169	0.0643							NS3	NS	LM			
7103	T	C	1.1%	174	0.0628		C	1.7%	1800	0.0853		NS3	S				
7127	C	T	1.1%	174	0.0628							NS3	S				
7138	A	G	1.2%	164	0.0659							NS3	NS	NS			
7147	T	C	1.1%	179	0.0613							NS3	NS	VA			
7150	T	C	1.6%	184	0.0653							NS3	NS	VA			
7165	C	G	1.0%	192	0.0579							NS3	NS	AG			
7169	G	T	1.6%	188	0.0819							NS3	S				
7183	G	T	1.1%	182	0.0605							NS3	NS	GV			
7199	G	A	1.2%	169	0.0643							NS4A	S				
7203	G	A	1.2%	162	0.0665							NS4A	NS	AT			
7221	T	A	1.2%	168	0.0646							NS4A	NS	FI			
7226	G						A	1.1%	1745	0.0675		NS4A	S				
7233	G	T	1.1%	181	0.0608							NS4A	NS	GC			
7239	C	A	1.0%	192	0.0579							NS4A	NS	QK			
7244	T	A	11.7%	188	0.3609		A	37.7%	2116	0.6624		NS4A	S				
7247	A	G	3.7%	189	0.1459		G	1.1%	2142	0.0635		NS4A	S				
7253	G	A	1.1%	186	0.0594							NS4A	S				
7254	A	G	1.1%	184	0.0600							NS4A	NS	RG			
7256	G	A	1.1%	185	0.0597							NS4A	S				
7259	T	A	1.1%	186	0.0594							NS4A	NS	HQ			
7277	T						C	4.6%	2165	0.1838		NS4A	S				
7328	T	C	1.3%	149	0.0712							NS4A	S				
7337	C						T	1.3%	2146	0.0644		NS4A	S				
7368	T	C	3.6%	165	0.1562							NS4A	S				

Ref Position	Ref Base	Serum Base	Serum SNP %	Serum Depth	Serum entropy		Culture Base	Culture SNP %	Culture Depth	Culture entropy		Features	S/NS	Residue change	Serum PCR call	P3 PCR call	P5 PCR call
7379	G	A	1.3%	159	0.0675							NS4A	S				
7416	T						A	4.3%	2355	0.1889		NS4B	NS				
7427	A	G	1.5%	134	0.0776							NS4B	S				
7436	G	T	7.2%	138	0.2962		T	36.8%	2351	0.6808		NS4B	S		G/t		K
7465	G						A	1.4%	1908	0.0761		NS4B	NS	RK			
7472	A	G	1.6%	122	0.0836							NS4B	S				
7475	C	T	1.6%	123	0.0831							NS4B	S				
7484	G	A	1.6%	125	0.0820							NS4B	S				
7485	T	G	1.6%	124	0.0826							NS4B	NS	FV			
7497	G	A	1.5%	130	0.0795							NS4B	NS	VI			
7519	T						C	1.8%	1634	0.0999		NS4B	NS	VA			T/c
7520	A	G	6.2%	146	0.2315		G	7.2%	1632	0.2586		NS4B	S				
7521												NS4B	NS	KE			A/g
7522	A						G	2.1%	1608	0.1001		NS4B	NS	KR			
7524												NS4B	NS	KR			A/g
7527	T						A	1.1%	1637	0.0632		NS4B	NS	LI			
7547												NS4B	S				A/g
7550	T	A	2.7%	148	0.1243							NS4B	NS	DE			
7573	G	C	1.2%	165	0.0655							NS4B	NS	GA			
7579	G	T	1.2%	172	0.0634							NS4B	NS	WL			
7582	G	A	1.2%	173	0.0631							NS4B	NS	GE			
7589	T	C	1.2%	172	0.0634							NS4B	S				
7597	T	A	1.1%	190	0.0584							NS4B	NS	LH			
7613	T	C	1.0%	205	0.0549							NS4B	S				
7623	G	A	1.0%	207	0.0544							NS4B	NS	GR			
7625	G	A	1.0%	208	0.0542							NS4B	S				

Ref Position	Ref Base	Serum Base	Serum SNP %	Serum Depth	Serum entropy		Culture Base	Culture SNP %	Culture Depth	Culture entropy		Features	S/NS	Residue change	Serum PCR call	P3 PCR call	P5 PCR call
7634	A	G	1.4%	209	0.0752							NS4B	S				
7643	T	C	0.9%	216	0.0526							NS4B	S				
7652	G						A	4.1%	1695	0.1702		NS4B	S				
7662	C	G	0.9%	216	0.0526							NS4B	NS	LV			
7667	C	T	0.9%	214	0.0530							NS4B	S				
7670	C	T	3.3%	214	0.1440		T	1.4%	1623	0.0718		NS4B	S				
7694	C	T	0.9%	213	0.0532		T	4.0%	1699	0.1680		NS4B	S				
7697	C	A	22.6%	212	0.5349							NS4B	S		C/a		
7749	T	C	1.0%	210	0.0538							NS4B	NS	FL			
7760	T	C	10.5%	210	0.3354		C	5.0%	1716	0.1971		NS4B	S				
7762	C	A	1.0%	210	0.0538							NS4B	NS	SY			
7772	G	A	1.0%	210	0.0538							NS4B	S				
7802	G						A	1.4%	1741	0.0727		NS4B	S				G/a
7811	C	T	2.1%	194	0.1004							NS4B	S				
7815	C	T	8.0%	188	0.2782		T	2.7%	1732	0.1231		NS4B	S				
7846	A	G	1.1%	178	0.0616							NS4B	NS	YC			
7856	C	A	1.1%	179	0.0613							NS4B	S				
7862	A	G	2.4%	164	0.0973							NS4B	S				
7878	C	T	1.1%	182	0.0605							NS4B	S				
7895	T						C	1.2%	1820	0.0646		NS4B	S				
7899	A	G	1.1%	189	0.0587							NS4B	NS	TA			
7910	A	G	1.1%	188	0.0589							NS4B	S				
7922	C						A	2.2%	1671	0.1040		NS4B	S				
7923	C	A	2.8%	213	0.1283							NS4B	NS	PT			
7926	A	T	0.9%	215	0.0528							NS4B	NS	TS			
7934	G						A	2.4%	1756	0.1129		NS4B	S				

Ref Position	Ref Base	Serum Base	Serum SNP %	Serum Depth	Serum entropy		Culture Base	Culture SNP %	Culture Depth	Culture entropy		Features	S/NS	Residue change	Serum PCR call	P3 PCR call	P5 PCR call
7950	C	T	0.9%	219	0.0520							NS4B	S				
7955	C						T	1.3%	1664	0.0683		NS4B	S				
7981	C	T	0.6%	315	0.0385							NS4B	NS	SL			
7982	A	G	0.6%	313	0.0387							NS4B	S				
7991	A	G	0.9%	336	0.0510							NS4B	S				
8001	G	T	1.9%	324	0.0922							NS4B	NS	DY			
8003	T	C	3.2%	317	0.1401		C	1.7%	2765	0.0846		NS4B	S				
8018	G	A	6.6%	334	0.2428		A	3.5%	3706	0.1507		NS4B	S				
8030	A	C	1.2%	333	0.0544		C	4.2%	3680	0.1855		NS4B	S				
8042	A	G	1.8%	332	0.0904							NS4B	NS	IM			
8090	A						G	1.6%	3291	0.0803		NS4B	S				
8103	G						A	1.6%	3153	0.0828		NS4B	NS	AT			
8112	G	A	0.7%	295	0.0406							NS4B	NS	AT			
8114	C	A	0.7%	297	0.0404							NS4B	S				
8126	C						T	1.3%	3055	0.0684		NS4B	S				
8130	C	T	0.7%	295	0.0406							NS4B	STOP	Q*			
8132	G	C	0.7%	291	0.0411							NS4B	NS	QH			
8142	T	C	0.7%	289	0.0413							NS4B	S				
8145	C	T	0.7%	289	0.0413							NS4B	NS	LF			
8162	A	G	3.0%	263	0.1362							NS4B	S				
8175	G	A	0.8%	260	0.0451							NS4B	NS	DN			
8183	A	G	0.8%	258	0.0454							NS4B	S				
8188	C	T	1.0%	198	0.0565							NS4B	NS	TM			
8189	G	A	1.0%	201	0.0558							NS4B	S				
8195	G	A	2.0%	196	0.0996		A	1.5%	2094	0.0848		NS4B	S				
8201	T	C	1.4%	145	0.0728							NS4B	S				

Ref Position	Ref Base	Serum Base	Serum SNP %	Serum Depth	Serum entropy		Culture Base	Culture SNP %	Culture Depth	Culture entropy		Features	S/NS	Residue change	Serum PCR call	P3 PCR call	P5 PCR call
8211	C						A	1.0%	864	0.0589		NS4B	NS	PT			
8223	A						C	1.4%	292	0.0606		NS4B	NS	IL			
8232	T						C	1.8%	109	0.0915		NS4B	S				
8236	T	A	2.2%	135	0.1066							NS4B	NS	FY			
8257	G	A	1.7%	120	0.0848							NS4B	NS	GD			
8258	T	C	1.7%	119	0.0853							NS4B	S				
8264	C	T	24.3%	136	0.6573		T	6.3%	347	0.2362		NS4B	S		C/t		
8270	A	T	1.6%	127	0.0810							NS4B	NS	RS			
8330	G	A	1.2%	165	0.0655							NS4B	S				
8341	G	A	1.2%	162	0.0665							NS4B	NS	GD			
8357	A	G	2.4%	165	0.1141							NS4B	S				
8358	C	A	1.2%	167	0.0649							NS4B	NS	LM			
8375	T						C	1.7%	1397	0.0868		NS4B	S				
8378	C	T	2.4%	168	0.1125							NS4B	S				
8399	A	G	1.3%	157	0.0682							NS4B	S				
8442	T	C	2.4%	166	0.1136							NS5A	S				
8460	A	G	2.5%	158	0.1181							NS5A	NS	KE			
8476	G	A	1.3%	149	0.0712							NS5A	NS	GE			
8480	G	C	1.3%	150	0.0708							NS5A	S				
8483	G	A	1.3%	149	0.0712							NS5A	S				
8485	A	G	1.4%	147	0.0720							NS5A	NS	KR			
8489	A	G	2.7%	146	0.1256		G	1.7%	1798	0.0814		NS5A	NS	IM			
8535	G						A	4.7%	1733	0.1888		NS5A	NS	GS			
8549	C	T	1.4%	139	0.0753							NS5A	S				
8553	T	G	1.4%	139	0.0753							NS5A	NS	LV			
8561	A						T	1.6%	1614	0.0873		NS5A	S				

Ref Position	Ref Base	Serum Base	Serum SNP %	Serum Depth	Serum entropy		Culture Base	Culture SNP %	Culture Depth	Culture entropy		Features	S/NS	Residue change	Serum PCR call	P3 PCR call	P5 PCR call
8572	T	C	5.2%	134	0.2051							NS5A	NS	LS			
8584	T	C	11.5%	130	0.3576		C	1.4%	1760	0.0763		NS5A	NS	IT			
8591	C	T	2.1%	140	0.0819							NS5A	S				
8618	C						A	1.5%	1985	0.0762		NS5A	S				
8627	A	G	9.9%	162	0.3224							NS5A	S				
8633	T						C	1.5%	2074	0.0745		NS5A	S				
8634	A	G	1.8%	164	0.0913							NS5A	NS	KE			
8636	A						G	1.3%	2073	0.0702		NS5A	S				
8637	C						T	1.1%	2072	0.0635		NS5A	NS	LF			
8639	T	C	1.2%	166	0.0652							NS5A	S				
8645	G						A	5.7%	1993	0.2192		NS5A	S				
8666	C	T	2.0%	151	0.0975							NS5A	S				
8669	G						T	1.9%	1889	0.0968		NS5A	S				
8674	A	G	38.7%	150	0.6672		G	3.5%	1872	0.1526		NS5A	NS	KR	A/g		A/g
8677	A	G	1.4%	145	0.0728							NS5A	NS	NS			
8689	G	C	1.3%	158	0.0679							NS5A	NS	RT			
8693	G						A	1.4%	1829	0.0704		NS5A	S				
8694	C	A	2.5%	163	0.0978							NS5A	NS	PT			
8698	T	C	1.2%	166	0.0652							NS5A	NS	VA			
8723	T	C	1.3%	156	0.0686							NS5A	S				
8746	A	G	1.2%	166	0.0652							NS5A	NS	KR			
8750	A	G	2.4%	164	0.1147							NS5A	S				
8771	A						G	1.2%	1880	0.0618		NS5A	S				
8780	C	T	1.1%	175	0.0625							NS5A	S				
8783	C	T	1.1%	176	0.0622							NS5A	S				
8787	A	T	1.1%	179	0.0613							NS5A	STOP	K*			

Ref Position	Ref Base	Serum Base	Serum SNP %	Serum Depth	Serum entropy		Culture Base	Culture SNP %	Culture Depth	Culture entropy		Features	S/NS	Residue change	Serum PCR call	P3 PCR call	P5 PCR call
8816	T	C	1.2%	164	0.0659		C	5.3%	1456	0.2356		NS5A	S				
8820	G	A	1.2%	164	0.0659							NS5A	NS	GR			
8821	G	A	1.2%	166	0.0652							NS5A	NS	GE			
8827	C	A	1.8%	169	0.0892		A	1.9%	1509	0.1027		NS5A	NS	TK			
8828	A	G	1.2%	169	0.0643							NS5A	S				
8829	C	A	1.2%	168	0.0646							NS5A	NS	LI			
8831	C	G	1.2%	166	0.0652							NS5A	S				
8843	T						C	3.1%	1513	0.1368		NS5A	S				
8848	G	T	1.2%	166	0.0652							NS5A	NS	WL			
8868	A	T	1.2%	162	0.0665							NS5A	NS	IL			
8876	G						A	4.2%	1885	0.1728		NS5A	S				
8879	G	A	1.2%	161	0.0669							NS5A	S				
8886	A	G	1.3%	159	0.0675							NS5A	NS	RG			
8891	C	T	1.3%	158	0.0679							NS5A	S				
8912	C	T	1.2%	166	0.0652							NS5A	S				
8915	A	G	1.2%	167	0.0649							NS5A	S				
8963	T	A	2.2%	136	0.0839							NS5A	STOP	C*			
8964	G	C	1.5%	135	0.0771							NS5A	NS	AP			
8981	C	A	1.5%	137	0.0762		A	1.9%	1870	0.0985		NS5A	NS	NK			
8983	C						A	1.3%	1837	0.0746		NS5A	NS	TK			
8984	A	T	3.1%	130	0.1374		T	3.7%	1829	0.1740		NS5A	S				
8987	G						A	1.4%	1753	0.0765		NS5A	S				
9001	T						A	1.5%	1632	0.0862		NS5A	NS	MK			
9026	C	T	3.2%	156	0.1418		T	1.0%	1726	0.0559		NS5A	S				
9035	T						C	4.2%	1724	0.1741		NS5A	S				
9039	T	A	1.3%	157	0.0682							NS5A	NS	ST			

Ref Position	Ref Base	Serum Base	Serum SNP %	Serum Depth	Serum entropy		Culture Base	Culture SNP %	Culture Depth	Culture entropy		Features	S/NS	Residue change	Serum PCR call	P3 PCR call	P5 PCR call
9056	T						C	1.2%	1604	0.0577		NS5A	S				
9059	T	C	8.5%	142	0.2896							NS5A	S				
9073	G	A	1.5%	137	0.0762							NS5A	NS	RK			
9079	A						C	1.1%	1516	0.0585		NS5A	NS	NT			
9083	T	C	1.5%	132	0.0785							NS5A	S				
9089	G						A	2.9%	1288	0.1430		NS5A	S				
9093	G	A	2.3%	133	0.1078		A	3.2%	1392	0.1425		NS5A	NS	EK			
9098	A						C	2.5%	1664	0.1178		NS5A	S				
9140	G						A	1.1%	2229	0.0599		NS5A	S				
9150	A	G	1.5%	197	0.0788		G	3.1%	2281	0.1386		NS5A	NS	MV			
9160	T	A	1.0%	193	0.0577							NS5A	NS	IK			
9165	C	T	1.0%	203	0.0553							NS5A	NS	PS			
9170	G						A	1.0%	2391	0.0591		NS5A	S				
9171	C						T	1.7%	2399	0.0852		NS5A	S				
9182	A	G	2.3%	215	0.1105							NS5A	S				
9183	G	A	0.9%	215	0.0528							NS5A	NS	VI			
9188	C	T	0.9%	217	0.0524							NS5A	S				
9199	T	C	0.9%	222	0.0514							NS5A	NS	VA			
9209	C	T	0.9%	235	0.0490							NS5A	S				
9210	A						T	2.8%	2757	0.1275		NS5A	NS	SC			
9225	G	A	2.1%	236	0.1026							NS5A	NS	VI			
9239	A	G	1.7%	233	0.0868							NS5A	S				
9248	T	C	16.6%	235	0.4494		C	8.1%	2829	0.2819		NS5A	S		T/c		
9267	A						G	1.4%	3026	0.0695		NS5A	NS	RG			
9269	G	A	3.1%	255	0.1395							NS5A	S				
9271	G	A	0.8%	256	0.0457							NS5A	NS	GE			

Ref Position	Ref Base	Serum Base	Serum SNP %	Serum Depth	Serum entropy		Culture Base	Culture SNP %	Culture Depth	Culture entropy		Features	S/NS	Residue change	Serum PCR call	P3 PCR call	P5 PCR call
9272	A	G	0.8%	255	0.0458							NS5A	S				
9275	C	T	1.6%	255	0.0807							NS5A	S				
9293	G	A	15.7%	254	0.4355							NS5A	S		G/a		
9311	G	A	1.7%	232	0.0871							NS5A	S				
9313	T	A	1.7%	233	0.0868							NS5A	NS	IK			
9315	G	A	0.9%	233	0.0494							NS5A	NS	EK			
9323	C	T	0.9%	219	0.0520							NS5A	S				
9324	G	T	0.9%	219	0.0520							NS5A	NS	AS			
9337	A	G	1.9%	215	0.0926							NS5A	NS	QR			
9343	C	T	0.9%	212	0.0534							NS5A	NS	TM			
9353	T	C	3.6%	221	0.1557							NS5A	S				
9362	T						A	1.5%	2223	0.0772		NS5A	NS	DE			
9374	C	A	0.9%	222	0.0514							NS5A	STOP	Y*			
9381	C	T	1.8%	227	0.0886							NS5A	NS	PS			
9383	T	C	2.2%	228	0.1202		C	2.8%	2313	0.1446		NS5A	S				
9387	C	A	1.3%	231	0.0693							NS5A	NS	LM			
9392	G	A	0.9%	227	0.0505		A	5.8%	2252	0.2236		NS5A	S				
9396	A	G	0.9%	233	0.0494							NS5A	NS	ND			
9406	T	A	2.7%	225	0.0979							NS5A	NS	VE			
9407	G	A	0.9%	225	0.0508							NS5A	S				
9411	G						A	1.1%	2081	0.0655		NS5A	NS	EK			
9415	T						A	1.1%	2059	0.0591		NS5A	NS	IK			
9430	C						T	1.1%	2056	0.0614		NS5A	NS	AV			
9437	T						A	10.9%	2162	0.3501		NS5A	S				T/a
9443	T						C	4.2%	2206	0.1747		NS5A	S				
9449	C	T	0.9%	233	0.0494							NS5A	S				

Ref Position	Ref Base	Serum Base	Serum SNP %	Serum Depth	Serum entropy		Culture Base	Culture SNP %	Culture Depth	Culture entropy		Features	S/NS	Residue change	Serum PCR call	P3 PCR call	P5 PCR call
9472	C	T	0.9%	227	0.0505							NS5A	NS	SL			
9480	G	T	0.8%	251	0.0464							NS5A	NS	AS			
9482	A	T	0.8%	253	0.0461							NS5A	S				
9489	G	A	0.8%	255	0.0458							NS5A	NS	AT			
9491	T	C	0.8%	256	0.0457							NS5A	S				
9509	T	C	25.2%	258	0.5645							NS5A	S		T/c		
9515	T	C	1.6%	252	0.0815							NS5A	S				
9516	G						A	2.6%	2190	0.1178		NS5A	NS	GS			
9517	G	A	0.8%	253	0.0461							NS5A	NS	GD			
9518	T	C	0.8%	252	0.0463							NS5A	S				
9519	A						G	3.1%	2189	0.1368		NS5A	NS	ND			
9546	G	A	0.8%	266	0.0443							NS5A	NS	AT			
9577	G	A	0.7%	296	0.0405							NS5A	NS	RK			
9582	A	T	0.7%	299	0.0402							NS5A	NS	IF			
9585	G	A	0.7%	301	0.0399							NS5A	NS	DN			
9594	C	A	0.7%	304	0.0396							NS5A	NS	LI			
9596	A	G	1.3%	303	0.0702							NS5A	S				
9602	A	G	1.0%	307	0.0425							NS5A	S				
9607	T	A	1.0%	303	0.0430							NS5A	NS	VD			
9618	G	A	0.7%	306	0.0394							NS5A	NS	GR			
9626	C						T	1.2%	2550	0.0630		NS5A	S				
9641	C	A	0.7%	283	0.0420							NS5A	S				
9660	G	C	0.7%	287	0.0416							NS5A	NS	GR			
9663	A	G	0.7%	281	0.0423							NS5A	NS	KE			
9671	A	G	0.7%	282	0.0422							NS5A	S				
9718	A	G	0.9%	222	0.0514		G	1.9%	2281	0.1001		NS5A	NS	EG			

Ref Position	Ref Base	Serum Base	Serum SNP %	Serum Depth	Serum entropy		Culture Base	Culture SNP %	Culture Depth	Culture entropy		Features	S/NS	Residue change	Serum PCR call	P3 PCR call	P5 PCR call
9721	G						A	1.1%	2232	0.0578		NS5A	NS	RK			
9724	A	C	2.6%	194	0.1055							NS5A	NS	QP			
9727	T	C	1.0%	193	0.0577		C	1.1%	2078	0.0683		NS5A	NS	VA			
9764	G	A	1.9%	208	0.0950							NS5A	S				
9789	G						A	1.1%	2122	0.0586		NS5A	NS	DN			
9791	T						G	1.4%	2134	0.0724		NS5A	NS	DE			
9802	T	A	1.1%	187	0.0592							NS5A	NS	LQ			
9814	T	C	3.1%	196	0.1369							NS5A	NS	VA			
9837	A	T	0.9%	213	0.0532							NS5A	STOP	K*			
9854	G	A	1.0%	192	0.0579							NS5A	S				
9865	G	A	2.8%	251	0.1167		A	5.9%	2091	0.2309		NS5A	NS	RK			
9881	A	G	0.8%	258	0.0454							NS5A	S				
9893	G	A	0.7%	307	0.0393							NS5A	S				
9923	G	A	5.1%	352	0.2018		A	2.6%	2355	0.1294		NS5A	S				
9951	A	C	1.0%	395	0.0566		C	3.6%	3373	0.1576		NS5B	NS	TP			
9952	C	T	1.0%	404	0.0555		T	2.7%	3442	0.1280		NS5B	NS	TI			
9953	C						A	1.1%	3431	0.0612		NS5B	S				
9957	C	T	1.0%	412	0.0547							NS5B	S				
9962	T						G	3.2%	3467	0.1586		NS5B	NS	FL			
9966	G						A	1.8%	3494	0.0965		NS5B	NS	EK			
9970	T						G	1.2%	3459	0.0659		NS5B	NS	LR			
9986	G	T	1.1%	435	0.0705							NS5B	S				
9987	C	T	1.1%	438	0.0701							NS5B	NS	PS			
9988	C	A	0.7%	449	0.0401							NS5B	NS	PH			
9998	G	T	0.7%	461	0.0393							NS5B	NS	KN			
10001	C	T	0.9%	463	0.0497							NS5B	S				

Ref Position	Ref Base	Serum Base	Serum SNP %	Serum Depth	Serum entropy		Culture Base	Culture SNP %	Culture Depth	Culture entropy		Features	S/NS	Residue change	Serum PCR call	P3 PCR call	P5 PCR call
10015	T	C	0.6%	468	0.0388							NS5B	NS	MT			
10052	G	A	0.7%	454	0.0398		A	3.1%	4125	0.1439		NS5B	S				
10058	T	C	0.7%	451	0.0400							NS5B	S				
10123	A	G	0.8%	359	0.0483							NS5B	NS	YC			
10127	G	T	1.7%	229	0.0880							NS5B	S				
10142	C	T	1.0%	205	0.0549							NS5B	S				
10148	A						G	2.0%	2507	0.0943		NS5B	S				
10160	G	A	1.5%	195	0.0795							NS5B	S				
10167	A	T	1.1%	190	0.0584							NS5B	NS	RW			
10180	C						T	1.4%	2392	0.0759		NS5B	NS	TI			
10182	G	A	1.1%	181	0.0608							NS5B	NS	VI			
10210	T	A	2.0%	152	0.0970		A	1.4%	2361	0.0803		NS5B	NS	LH			
10238	C	T	6.6%	152	0.2426		T	25.6%	2400	0.5691		NS5B	S		C/t	Y	C/t
10254	C	A	15.7%	140	0.4349							NS5B	NS	LI	C/a		
10286	C	T	2.8%	145	0.1262							NS5B	S				
10298	T	C	4.9%	143	0.1954							NS5B	S		T/c		
10322	G						A	2.0%	2293	0.0983		NS5B	S				
10325	C	A	1.3%	149	0.0712							NS5B	S				
10340	A	G	1.1%	174	0.0628							NS5B	S				
10341	A	G	1.1%	175	0.0625							NS5B	NS	SG			
10346	A	G	1.6%	186	0.0647							NS5B	S				
10349	T	G	1.6%	187	0.0822							NS5B	S				
10353	A	T	1.0%	194	0.0574							NS5B	NS	RW			
10355	G	A	1.5%	196	0.0620							NS5B	S				
10359	G	A	2.4%	207	0.1001							NS5B	NS	EK			
10364	A	G	0.9%	212	0.0534							NS5B	S				

Ref Position	Ref Base	Serum Base	Serum SNP %	Serum Depth	Serum entropy		Culture Base	Culture SNP %	Culture Depth	Culture entropy		Features	S/NS	Residue change	Serum PCR call	P3 PCR call	P5 PCR call
10365	T	C	0.9%	212	0.0534							NS5B	S				
10379	G	A	1.4%	213	0.0740							NS5B	S				
10382	C						T	12.0%	2393	0.3663		NS5B	S				C/t
10399	G						A	5.9%	2333	0.2246		NS5B	NS	SN			
10409	G	A	0.8%	250	0.0466		A	2.4%	2130	0.1117		NS5B	S				
10467	A	G	1.1%	380	0.0584							NS5B	NS	KE			
10470	T	C	1.3%	385	0.0604							NS5B	S				
10473	T						C	1.2%	3390	0.0674		NS5B	S				
10497	C	T	0.8%	391	0.0450							NS5B	NS	HY			
10508	G	C	1.0%	400	0.0629							NS5B	S				
10553	G	A	3.8%	395	0.1615		A	4.4%	3444	0.1860		NS5B	S				
10568	G	A	1.5%	399	0.0780							NS5B	S				
10573	C	T	1.0%	384	0.0579							NS5B	NS	AV			
10598	C						A	1.7%	3458	0.0843		NS5B	S				
10610	G	A	1.4%	361	0.0730							NS5B	S				
10625	C						T	1.6%	3678	0.0869		NS5B	S				
10634	A	G	1.2%	325	0.0664							NS5B	S				
10638	T	C	1.2%	322	0.0559							NS5B	S				
10641	G	A	0.9%	321	0.0409		A	1.9%	3707	0.0934		NS5B	NS	VI			
10643	C						T	1.0%	3720	0.0560		NS5B	S				
10645	G	A	0.9%	320	0.0411							NS5B	NS	RK			
10646	G	T	1.3%	319	0.0674							NS5B	NS	RS			
10653	A	G	0.6%	309	0.0391							NS5B	NS	KE			
10658	C	T	1.7%	299	0.0850							NS5B	S				
10659	G	T	1.4%	292	0.0819							NS5B	NS	GW			
10676	T	C	1.0%	311	0.0544							NS5B	S				

Ref Position	Ref Base	Serum Base	Serum SNP %	Serum Depth	Serum entropy		Culture Base	Culture SNP %	Culture Depth	Culture entropy		Features	S/NS	Residue change	Serum PCR call	P3 PCR call	P5 PCR call
10679	T	A	1.0%	311	0.0421							NS5B	STOP	Y*			
10696	A	C	0.8%	265	0.0444							NS5B	NS	KT			
10697	G						A	2.5%	2071	0.1290		NS5B	S				
10700	C	T	1.5%	272	0.0766		T	3.0%	2067	0.1389		NS5B	S				
10703	G						A	2.0%	2081	0.1104		NS5B	S				
10706	A	G	0.7%	291	0.0411							NS5B	S				
10708	G						A	2.3%	2114	0.1071		NS5B	NS	RK			
10712	T	C	1.0%	297	0.0565							NS5B	S				
10715	T						C	1.3%	2054	0.0667		NS5B	S				
10721	C	T	1.0%	293	0.0571		T	1.0%	2009	0.0563		NS5B	S				
10724	C	T	1.0%	294	0.0441							NS5B	S				
10740	C	T	0.9%	343	0.0502							NS5B	S				
10781	G	T	0.8%	489	0.0475							NS5B	NS	ED			
10799	C						T	2.7%	3104	0.1297		NS5B	S				C/t
10820	T	C	4.1%	484	0.1721		C	2.3%	3133	0.1123		NS5B	S				
10839	G	A	0.9%	469	0.0491							NS5B	NS	VI			
10853	A	G	1.5%	474	0.0700		G	7.0%	2901	0.2575		NS5B	S				
10859	A						G	1.9%	2822	0.0986		NS5B	S				
10872	T	C	1.0%	477	0.0582							NS5B	S				
10886	T	C	1.5%	468	0.0707							NS5B	S				
10889	T	A	1.3%	462	0.0693							NS5B	NS	NK			
10890	A	T	1.3%	461	0.0694							NS5B	STOP	K*			
10910	T	C	1.0%	401	0.0559							NS5B	S				
10918	A						T	1.1%	2578	0.0645		NS5B	NS	NI			
10931	C	T	0.8%	381	0.0460							NS5B	S				
10943	T	C	0.8%	375	0.0466							NS5B	S				

Ref Position	Ref Base	Serum Base	Serum SNP %	Serum Depth	Serum entropy		Culture Base	Culture SNP %	Culture Depth	Culture entropy		Features	S/NS	Residue change	Serum PCR call	P3 PCR call	P5 PCR call
10997	T						C	2.6%	1898	0.1379		NS5B	S				T/c
11007	T	A	2.2%	185	0.1043							NS5B	NS	YN			
11009	C	A	1.8%	166	0.0904							NS5B	STOP	Y*			
11012	C	A	2.6%	151	0.1223							NS5B	STOP	Y*			
11015	T						A	2.6%	1409	0.1412		NS5B	STOP	Y*			
11030	C	T	1.3%	158	0.0679							NS5B	S				
11045	C						T	5.4%	1539	0.2099		NS5B	S				
11070	C	T	1.1%	180	0.0610							NS5B	NS	PS			
11107	A	T	1.0%	207	0.0544							NS5B	NS	NI			
11108	C	T	44.1%	213	0.6862		T	5.7%	1497	0.2199		NS5B	S		C/t		C/t
11129	G	A	1.7%	235	0.0862							NS5B	S				
11130	C	T	0.8%	236	0.0489							NS5B	NS	PS			
11131	C	A	0.8%	237	0.0487							NS5B	NS	PQ			
11162	T	C	0.8%	261	0.0450							NS5B	S				
11165	C	A	0.8%	264	0.0445							NS5B	S				
11168	A	G	0.8%	265	0.0444							NS5B	S				
11187	T	C	0.8%	252	0.0463							NS5B	NS	CR			
11189	C	T	1.2%	256	0.0638							NS5B	S				
11202	A	G	1.5%	263	0.0788							NS5B	NS	IV			
11204	C	T	1.5%	266	0.0780							NS5B	S				
11207	A	G	0.7%	268	0.0440							NS5B	S				
11210	C	T	0.7%	270	0.0437							NS5B	S				
11219	C	T	1.9%	263	0.1070		T	1.2%	1893	0.0693		NS5B	S				
11224	G	T	0.7%	267	0.0441							NS5B	NS	RM			
11235	A	T	0.8%	262	0.0448							NS5B	NS	IF			
11243	C	T	0.7%	270	0.0437							NS5B	S				

Ref Position	Ref Base	Serum Base	Serum SNP %	Serum Depth	Serum entropy		Culture Base	Culture SNP %	Culture Depth	Culture entropy		Features	S/NS	Residue change	Serum PCR call	P3 PCR call	P5 PCR call
11246	T	C	0.8%	266	0.0443							NS5B	S				
11252	C	T	0.8%	250	0.0466							NS5B	S				
11254	A	T	0.8%	248	0.0469							NS5B	NS	DV			
11259	T	C	0.8%	256	0.0457							NS5B	NS	FL			
11262	T	C	0.8%	254	0.0460							NS5B	S				
11266	T	C	0.8%	254	0.0460							NS5B	NS	IT			
11273	G						A	1.4%	2161	0.0806		NS5B	S				
11295	G	T	0.8%	246	0.0472							NS5B	NS	AS			
11306	G	A	0.7%	273	0.0433							NS5B	S				
11309	G	A	0.7%	272	0.0434							NS5B	NS	MI			
11314	T	G	0.7%	272	0.0434							NS5B	NS	IS			
11328	G	T	0.8%	261	0.0450							NS5B	NS	GC			
11339	A	G	0.7%	267	0.0441							NS5B	S				
11348	G						A	3.0%	2890	0.1351		NS5B	S				
11351	A						G	1.3%	2855	0.0715		NS5B	S				
11355	G						A	1.6%	2847	0.0827		NS5B	NS	EK			
11357	A	G	7.8%	230	0.2745		G	28.3%	2830	0.5958		NS5B	S		A/g	A/g	A/g
11360	A	G	0.9%	229	0.0501							NS5B	S				
11362	T						A	1.2%	2777	0.0723		NS5B	NS	MK			
11369	T						A	4.4%	2813	0.1854		NS5B	S				
11372	C	T	0.9%	231	0.0497							NS5B	S				
11393	G	T	1.0%	207	0.0544							NS5B	NS	ED			
11444	T	A	0.8%	243	0.0477							NS5B	NS	SR			
11451	G	T	0.7%	273	0.0433							NS5B	NS	AS			
11515	G	A	1.1%	358	0.0613							NS5B	NS	RK			
11519	T	C	1.0%	383	0.0484							NS5B	NS	SW			

Ref Position	Ref Base	Serum Base	Serum SNP %	Serum Depth	Serum entropy		Culture Base	Culture SNP %	Culture Depth	Culture entropy		Features	S/NS	Residue change	Serum PCR call	P3 PCR call	P5 PCR call
11531	C						T	4.8%	2812	0.1929		NS5B	S				
11571	T						A	3.5%	4138	0.1541		NS5B	NS	WR			
11579	G	A	1.0%	573	0.0648							NS5B	S				
11584	T	C	1.4%	572	0.0736							NS5B	NS	VA			
11606	C	T	1.6%	569	0.0813							NS5B	S				
11629	C	T	0.9%	568	0.0504							NS5B	NS	AV			
11630	G	A	0.9%	567	0.0505							NS5B	S				
11636	A	G	0.7%	558	0.0425		G	6.1%	3970	0.2291		NS5B	S				
11639	A	G	0.7%	556	0.0427							NS5B	S				
11681	T	C	1.0%	521	0.0541		C	1.6%	3863	0.0798		NS5B	S				
11702	T	C	1.1%	528	0.0554							NS5B	S				
11765	G	A	0.9%	429	0.0529							NS5B	S				
11786	C						T	4.5%	2482	0.1900		NS5B	S				
11797	G	A	0.8%	247	0.0471							NS5B	NS	RK			
11798	A	G	1.6%	249	0.0823							NS5B	S				
11800	T	A	0.8%	252	0.0463		A	1.6%	2288	0.0924		NS5B	NS	IK			
11807	G	T	2.7%	261	0.1396							NS5B	NS	QH			
11820	G	A	1.2%	256	0.0638							NS5B	NS	VI			
11828	G	A	1.7%	241	0.0845							NS5B	S				
11832	G						A	1.1%	2297	0.0600		NS5B	NS	EK			
11844	C						A	4.9%	2518	0.1955		NS5B	NS	LI			
11884	A	T	0.7%	279	0.0425							NS5B	NS	HL			
11889	T	A	0.8%	266	0.0443							NS5B	NS	YN			
11897	T	C	8.5%	272	0.2898		C	27.2%	2539	0.5965		NS5B	S			T/c	
11900	C	T	1.1%	285	0.0584							NS5B	S				
11903	A	G	1.0%	287	0.0581							NS5B	S				

Ref Position	Ref Base	Serum Base	Serum SNP %	Serum Depth	Serum entropy		Culture Base	Culture SNP %	Culture Depth	Culture entropy		Features	S/NS	Residue change	Serum PCR call	P3 PCR call	P5 PCR call
11906	T						C	1.5%	2637	0.0786		NS5B	S				
11930	T						C	11.0%	2349	0.3457		NS5B	S			T/c	T/c
11936	A	G	0.6%	312	0.0388		G	1.5%	2325	0.0781		NS5B	S				
11937	C						T	2.1%	2328	0.1037		NS5B	S				
11939												NS5B	S				A/g
11957	G	A	0.8%	396	0.0445							NS5B	S				
11993	G	A	1.1%	446	0.0534							NS5B	S				
11996	A						G	1.9%	2048	0.0909		NS5B	S				A/g
11999	T	C	0.9%	446	0.0512							NS5B	S				
12010	A						C	2.5%	1943	0.1144		NS5B	NS	NT			
12012	A	G	1.9%	423	0.0866							NS5B	NS	IV			
12029	A						G	1.8%	1742	0.0959		NS5B	S				
12033	G	A	1.2%	431	0.0550							NS5B	NS	VI			
12035	C	T	0.7%	441	0.0407							NS5B	S				
12079	T						C	1.7%	3770	0.0867		UTR	NA				T/c
12084	T	C	5.5%	656	0.2097							UTR	NA				
12093	G	A	2.3%	652	0.1095		A	1.7%	3967	0.0839		UTR	NA				
12095	T	C	4.0%	648	0.1683		C	5.0%	3965	0.2021		UTR	NA				
12107	C	T	2.6%	618	0.1202							UTR	NA				
12110	A	G	1.0%	614	0.0612							UTR	NA				
12112	T	C	1.8%	611	0.0933							UTR	NA				
12114	T	C	1.1%	610	0.0627		C	3.7%	3789	0.1657		UTR	NA				
12116	T						C	2.8%	3788	0.1328		UTR	NA				
12133	T	C	1.2%	590	0.0649							UTR	NA				
12176	C	T	3.4%	532	0.1478		T	3.4%	3425	0.1522		UTR	NA				
12178	G	A	0.8%	530	0.0444							UTR	NA				

Ref Position	Ref Base	Serum Base	Serum SNP %	Serum Depth	Serum entropy		Culture Base	Culture SNP %	Culture Depth	Culture entropy		Features	S/NS	Residue change	Serum PCR call	P3 PCR call	P5 PCR call
12193	T	A	0.6%	466	0.0389							UTR	NA				
12199	A						G	8.7%	3288	0.2958		UTR	NA				
12211	C	T	0.8%	364	0.0478							UTR	NA				
12223	A	T	0.9%	346	0.0384							UTR	NA				
12228	A	G	0.9%	340	0.0505							UTR	NA				
12238	T	G	0.9%	325	0.0405							UTR	NA				
12249	A	T	0.6%	313	0.0387							UTR	NA				
12279	C						T	2.3%	1043	0.1321		UTR	NA				
12280	C						A	1.3%	862	0.0683		UTR	NA				

NADL variant base sites

NADL position	MRI103 position	NADL Base	Variant Base	NADL SNP %	NADL Depth	NADL entropy	Features
25	10	T	A	2.1%	1056	0.1181	UTR
34	19	T	C	2.4%	1602	0.1207	UTR
1830	1816	G	A	6.3%	9383	0.2348	E1
2564	2549	C	A	2.9%	8143	0.1349	E2
4022	4007	A	T	3.8%	4759	0.1643	NS2-3
4068	4053	T	G	3.4%	4673	0.1529	NS2-3
5872	5588	A	G	3.7%	5148	0.1614	NS2-3
10838	10554	A	G	4.5%	15956	0.1932	NS5B
10869	10585	A	G	2.2%	13461	0.1138	UTR
10890	10606	T	G	3.9%	12493	0.1777	UTR
12293	12009	A	G	2.1%	12725	0.1018	UTR
12367	12082	A	G	2.4%	19659	0.1152	UTR
